# Seminal and testicular microbiome and male fertility: A systematic review

**DOI:** 10.1097/j.pbj.0000000000000151

**Published:** 2021-12-03

**Authors:** Pedro Brandão, Manuel Gonçalves-Henriques, Nathan Ceschin

**Affiliations:** a Department of Reproductive Medicine, Instituto Valenciano de Infertilidad, Valencia, Spain; b Faculdade de Medicina da Universidade do Porto, Porto, Portugal; c Department of Obstetrics and Gynecology, Hospital Prof. Doutor Fernando da Fonseca, Amadora, Lisbon, Portugal.

**Keywords:** assisted reproductive techniques, male infertility, microbiome, semen, testicle

## Abstract

Microbiome is of upmost importance for the well-being of the human body. Based on culture and PCR methods, seminal flora has been pointed as a potential cause for some of the unexplained male infertility.

This is a systematic review about the effect of seminal microbiota studied by Next Generation Sequencing techniques on sperm quality and male fertility, performed according to PRISMA statement.

Nine articles were included. Results of different studies are diverse. It seems that microbiota may a play a role in seminal quality and further male fertility, but the way this effect is modulated is still to be unknown. *Lactobacillus* spp seemed to play a beneficial role in semen quality, but the role of the remaining bacteria is unclear.

Due to the lack of research and the incongruence of the results so far, the effect of microbiota on seminal quality is still unclear.

## Abstract 2

Supplemental Digital Content is available in the text

## Introduction

Human microbiota consists of communities of bacteria, viruses and fungi. Its genetic load and its interaction with the surrounding environment form the microbiome. The study of human microbiome has been of great interest within the scientific community across all medical fields, in particular its role in modulating specific tissue functions.^1^

Nowadays, next-generation DNA sequencing (NGS) has enabled in-depth sample analysis. Sequencing 1 of 9 hypervariable regions (V1–V9) within the bacterial 16S rRNA gene allows the identification of bacteria from phyla to species level, even though most of the times its power of discrimination below genus level is inadequate. Resorting to public databases, one can proceed to a thorough description of specific bacterial taxa and their relative abundances, ultimately profiling samples microbiome in detail.^2–5^

Infertility affects around 10%–15% of the couples in reproductive age and male factors may be responsible for up to 40% of the cases. The gold standard tool for diagnosing and accessing male fertility is semen analysis, also called semino- gram.^6^ Based on a few main parameters—volume, concentration, spermatozoa motility and morphology—and reference criteria published by the World Health Organization (WHO) in 2010, sperm may be classified as oligozoospermia (<15 millions/mL), asthenozoospermia (total motility <40% or progressive motility <32%), teratozoospermia (normal specimens <4%), azoospermia (absence of spermatozoa) or a combination of them.^7,8^ Several factor may affect sperm quality and therefore male fertility, including lifestyle, medical disorders (endocrine, urologic, infectious, neurologic, etc), trauma, physical impairment, psychological issues, sexual disorders, chromosomal or genetic abnormalities.^9–11^ However, about 30%–70% of cases are idiopathic.^12,13^

Many factors have been pointed so far as possible causes for idiopathic male infertility, including genetics, epigenetics, proteomics, DNA fragmentation and microbiome.^14^

An incidence of asymptomatic bacteriospermia in infertile men of around 33% is estimated, even though incidences from 15% to 70% have also been found.^15,16^ Species most commonly isolated in sperm are *Enterobacteriaceae* (including *Escherichia coli, Enterococcus faecalis, Klebsiella* spp, *Salmonella* spp, *Proteus* spp and *Pseudomonas* spp), *Streptococcus* spp (*S agalactiae, S anginosus, S faecalis and S viridians*), *Staphylococcus* spp *(S aureus, S haemolyticus, S epidermidis)*, sexually transmitted infections (STI's) agents *(Ureaplasma urealyticum, Mycoplasma spp and Chlamydia trachomatis)*, *Gardnerella vaginalis, Bacter- oides* spp, *Morganella morganii*, and others. Bacteria in semen may impair both sperm motility and the acrosome reaction, may cause alterations in sperm morphology and may promote an inflammatory status through the production of reactive oxygen species (ROS).^17^ Even though bacteriospermia seems to deteriorate semen parameters, studies are not consistent and so the real impact of bacteria in semen quality is still not clear.^18–25^

Most research developed in this field is based on culture or PCR (protein chain reaction) methods, targeting specific agents. However, these methods have 2 important flaws—many bacteria may not be cultured or identified and they are limited to bacteria for which tests are directed—they do not scan the whole microbiota, unlike NGS.

Until date, unlike for the female genital tract, few data has been published based on NGS methods concerning seminal and testicular microbiota.^26^ This would be an interesting field of research, especially to evaluate the potential impact of the microbioma on male fertility, semen quality based on semen analysis and the outcomes of ART, such as quality of resulting embryos and clinical success measures, including pregnancy, miscarriage and live birth rates.

## Material and methods

The aim of this work is to review the effect of seminal and testicles’ microbiota studied by next generation sequencing techniques on sperm quality, male fertility and the outcomes of assisted reproductive treatments (ART) in humans.

A systematic review of all articles listed in Pubmed, SCOPUS and Cochrane Library was conducted in October 2020 using the query: (microbiome or microbiota or biofilm or 16s) and (semen or seminal or sperm or spermatozoa).

Original works about the subject published until date were included. Only research in humans and addressing semen analysis (seminogram) parameters, fertility or outcomes of ART were included. Reviews, case reports, case series, editorials, letters to the editor, comments, corrigenda, book chapters and works on animals were excluded. There were no language restrictions as all the articles requiring full text analysis were in languages the authors master. References of the selected articles were thoroughly reviewed in order to include other potentially related articles.

All process of study selection was performed independently by 2 reviewers (P.B. and M.G.H.). Inconsistencies between retrieved selections were resolved through discussion and agreement between both authors.

As a systematic review, there was no need for an ethical approval.

### Study appraisal

**Figure 1:**
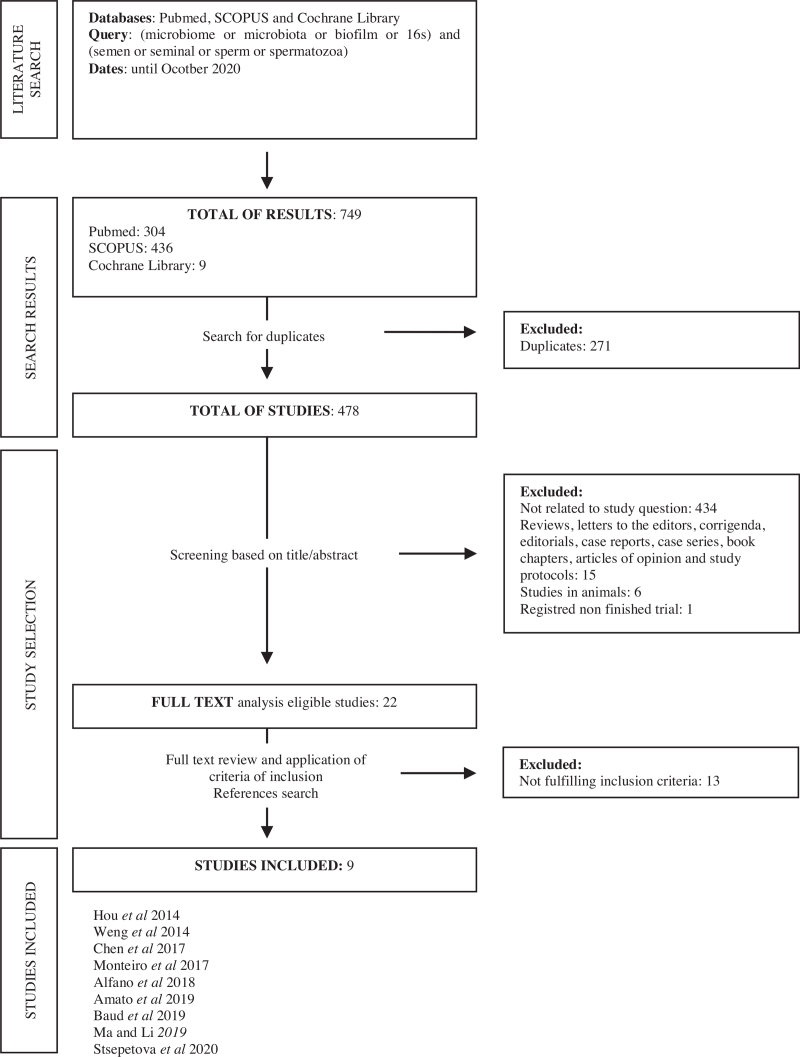
Flow diagram of study selection (according to PRISMA statement).

With the initial search using the query, a total of 749 results were retrieved (PubMed: 304, SCOPUS: 436, Cochrane Library: 9). Duplicates were removed (n = 271). All articles titles and/or abstracts were analyzed. Works not related to the study question (n = 434), studies in animals (n = 6), unfinished trials (n = 1), reviews, letters to the editors, corrigenda, editorials, case reports, case series, book chapters, articles of opinion and study protocols were excluded (n = 15). From the 22 articles retrieved, 13 were excluded after full text analyses either due to the absence of reference to the influence of microbiota in seminogram, fertility or ART outcomes, or studies based exclusively on culture methods or sequencing directed to specific bacteria and not the whole microbiota. Nine articles were selected. References search revealed no other studies to be included (Fig. 1).

This review will be divided in the following sections: description of seminal microbiota; the impact on fertility and seminal parameters of the following variables—richness and diversity of species, the role of *Lactobacillus* spp and the influence of different species; the impact of microbiota on ART outcomes; and the influence of testicular microbiota on sperm retrieval (SDC, Table 1, http://links.lww.com/PBJ/A9).

## Results

### Seminal microbiota description

The most common bacteria found in seminal microbiota belong to 4 phyla: Actinobacteria (*Corynebacterium* spp and *Rhodococcus* spp), Bacteroidetes (*Prevotella* spp), Firmicutes (*Anaerococcus* spp, *Bacillus* spp, *Finegoldia* spp, *Lactobacillus* spp, *Staphylococcus* spp, *Streptococcus* spp and *Veillonella* spp) and Proteobacteria (*Burkholderia* spp, *Haemophilus* spp, *Proteus* spp and *Pseudomonas* spp) (Table 1).

**Table 1:** Main seminal bacterial phyla and genera

PHYLUM	GENERUM
*Actinobacteria*	*Atopobium* spp^33^ *Corynebacterium* spp^28–30,33^ *Gardnerella* spp^31^ *Propionibacterium* spp^28^ *Rhodococcus* spp^27^
*Bacteroidetes*	*Cloacibacterium* spp^33^ *Porphyromonas* spp^33^ *Prevotella* spp^27,29–31,33,34^
*Firmicutes*	*Aerococcus* spp^33^ *Anaerococcus* spp^28,33^ *Bacillus* spp^27^ *Clostridium* spp^33^ *Enterococcus* spp^28^ *Finegoldia* spp^30,31,33^ *Gemella* spp^33^ *Lactobacillus* spp^27,29–31,33,34^ *Peptoniphilus* spp^28,33^ *Staphylococcus* spp^27,28,30,33,34^ *Streptococcus* spp^29–31,33^ *Veillonella* spp^27,33^
*Proteobacteria*	*Acidovorax* spp^33^ *Bradyrhizobium* spp^33^ *Burkholderia* spp^30^ *Haemophilus* spp^30,31^ *Pelomonas* spp^33^ *Proteus* spp^27^ *Pseudomonas* spp^27,31^ *Ralstonia* spp^33^ *Rhodanobacter* spp^31^
Other	*Ureaplasma* spp^33^

The proportion of each bacterium, though, varied considerably between samples and studies.^27–30^

### Richness and diversity of species

Data concerning the relation of seminal richness and diversity of species and fertility were incongruous.

Chen et al compared fertile controls with patients with azoospermia, both obstructive (OA) and non-obstructive (NOA). They found a progressively lower number of species in infertile patients’ semen (average number of operative taxonomic units (OTU): controls 1093, OA 925 and NOA 840).^27^ Amato et al observed a higher diversity in sperm of infertile (idiopathic infertility) patients (SDI: 4.3–4.8 infertile vs 3.1 controls; *P* = .004).^29^ Monteiro et al found progressively higher rates of richness and diversity of species from controls to patients with asthenozoospermia, oligoas- thenozoospermia and seminal hyperviscosity.^28^

On the other hand, Weng et al found no differences in richness (Chao index, *P* = .08) or diversity (SDI, *P* = .33) of species between infertile patients when compared to controls.^31^ Nested on the same samples, a later study corroborated these findings.^32^ Baud et al also observed no differences in richness and diversity of species between normosperic and abnormal seminogram group (P > .05) except a minor increase in Chao1 index in the group with abnormal motility (*P* = .02).

### Lactobacillus spp

*Lactobacillus* spp revealed a positive association with male fertility and seminal parameters.

Weng et al found higher rates of this genus in healthy controls (in particular *L crispatus* and *L acidophilus*).^31^

A lower amount of *Lactobacillus* spp was found in azoospermic patients (Controls 6.79%, OA 17.98 and NOA 17.24%), as well as patients with oligoasthenozoospermia, teratozoospermia or seminal hyperviscosity.^27,28,30^

### Influence of different species

At the phyla level, Chen et al found that *Bacteroidetes* and *Firmicutes* predominated in azoospermic patients and *Proteobacteria* and *Actinobacteria* in fertile patients.^27^

At the family level, Amato et al found no differences between infertile patients and controls concerning different families of bacteria.^29^

At the genus level, Baud et al and Weng et al found an association between normal seminogram and *Propionibacterium* spp, *Gardnerella vaginalis, Atopobium vaginalis* and *Staphylococcus* spp, contradicting previous findings of culture and PCR based studies. *Prevotella* spp, especially *P bivia*, and *Haemophilus parainfluenza* were more frequent in samples with at least 1 criteria of low seminal quality.^30,31^

Chen et al found a dominance of *Sneathia* spp and *Lysobacter* spp (*P* < .05) in NOA patients and *Solibacillus* spp, *Campylo- bacteraceae* and *Plesiomonas* spp in OA patients (*P* < .05).^27^

Hou et al reported that *Anaerococcus* spp were more frequent in infertile patients (asthenozoospermic, oligozoospermic or azoospermic) (*P* = .0012) but no differences were found concerning other genera (*P* > .47).^33^

Monteiro et al reported that patients with oligoasthenozoo- spermia or seminal hyperviscosity had more bacteria of the genera *Pseudomonas* spp, *Klebsiella* spp, *Aerococcus* spp, *Actinobaculum* spp and *Neisseria* spp and less *Propionibacte- rium* spp.^28^ They also found *Corynebacterium* spp, *Haemophilus* spp and *Streptococcus* spp, classically associated with ISTs, in some patients with normal seminograms, suggesting that these entities may also be part of commensal flora.

Stsepetova et al reported that *Staphylococcus* spp, *Erysipelo- trichaceae* and *Bacteroidia* were associated with the presence of neutrophils in semen. *Staphylococcus* spp was detected only in patients with inflammation. *Bacteroidetes* and *Proteobacteria* were negatively associated with sperm motility.

*Escherichia coli* showed no relation with seminal parameters.^31^

### Sperm microbiota and ART outcomes

Amato et al compared results after intrauterine insemination (IUI) and found no differences according to sperm diversity or microbiota composition.^29^ Although not clearly specified, the authors of this study refer to successful IUI as clinical pregnancy (which is usually defined as the presence of intrauterine gestational sac detectable by ultrasound).

The quality of embryos resulting from IVF was found to be negatively affected by seminal *Proteobacteria* and *Corynebacte- rium* spp, while *Enterobacteriaceae* were correlated with better embryo quality, based on morphologic assessment on cleavage stage.^34^

No studies were found regarding clinical ART outcomes, such as such as implantation, clinical pregnancy, ongoing pregnancy, miscarriage and live birth rates.

### Testicular microbiota and retrieval of spermatozoa

Alfano et al compared microbiota of testicular tissue of men with idiopathic non-obstructive azoospermia (iNOA) with and without sperm retrieval after TESA (Testicular Sperm Aspiration) or TESE (Testicular Sperm Extraction) with men after orchiectomy for other purposes. Just like seminal microbiota, the most common phyla in testicular tissue of men with normal germ line were *Actinobacteria*, *Bacteroidetes*, *Firmicutes* and *Proteobacteria*. They observed an increase of dysbiosis in iNOA patients, with a higher amount of 16 s DNA copies reflecting a higher load of bacteria (*P* = .02) and lower richness and diversity of species, in particular due to greatly decreased amounts of *Bacteroidetes* and *Proteobacteria* (*P* = .00002). Therefore, iNOA patients had a testicular flora dominated by *Firmicutes* and *Actinobacteria*, the latter being the dominant phyla in patients with no sperm retrieval.^35^

## Discussion

Results of studies concerning the influence of microbiome on seminal quality and male fertility are quite inconsistent. Not only studies using NGS contradict each other in some points, but also they bring some information inconsistent with previous knowledge based on culture or PCR methods.

Semen microbiome was shown to be dominated by 4 phyla of bacteria—*Actinobacteria, Bacteroidetes, Firmicutes* and *Proteobacteria*. The proportion of each phyla and respective genus differed among different studies, as well as the impact of different proportions on seminal parameters.

Highly discordant data was found concerning richness and diversity of species between groups of fertile men and patients with abnormal seminal parameters. One study revealed a lower number of species in infertile patients, other studies found higher levels of richness and diversity of species within this population and other groups reported no relation at all between these factors and seminal parameters. No conclusion may be drawn based on these findings.

Just like what happens in the vagina, *Lactobacillus* spp seem to play a beneficial role in seminal health, all the 4 four studies focusing this genus reported better seminal analysis results associated with these bacteria, especially *L crispatus* and *L acidophilus*.

At the phyla level, one study reported results in semen and other in testicular tissue. *Firmicutes* revealed a consistent negative impact on both tissues. About *Proteobacteria*, 2 studies revealed a positive association with sperm quality both in semen and testis respectively, but another study reported a negative effect of these bacteria in sperm motility. *Bacteroidetes* were found to have a negative influence in semen quality, in particular sperm motility, but a positive one in testis. On the other hand, *Actinobacteria*, that shown positive association with seminal quality, revealed a negative relation in testis. Based on this, either the dominance of different phyla of bacteria may play different roles depending on the tissue, or studies have discordant results, so no conclusion may be drawn so far based on phyla.

At the genus level, *Propionibacterium* spp, *Atopobium vaginalis, Gardnerella vaginalis* and *Staphylococcus* spp were found by some authors to have a positive association with seminal quality. This is somehow intriguing, as according to previous knowledge based on culture or PCR methods, at least the last 2 had been reported to negatively affect sperm. Besides, *Staphylococcus* spp has also been reported by one study to be consistently associated with neutrophils and inflammation in semen. Some studies revealed that other genera and families, including *Actinobaculum* spp, *Aerococcus* spp, *Anaerococcus* spp, *Campylobacteraceae, Haemophilus* spp, *Klebsiella* spp, *Lysobacter* spp, *Neisseria* spp, *Plesiomonas* spp, *Prevotella* spp, *Pseudomonas* spp, *Sneathia* spp, and *Solibacillus* spp had a negative with one or various semen parameters. However, some other authors found no significant association of many of these and other bacteria with seminal quality.

Only 2 groups make reference to the association between seminal microbiota and ART outcomes. One group reported no apparent effect on IUI results. The other group evaluated the quality of the resulting embryos after *in vitro* fertilization and it was negatively affected by seminal *Proteobacteria* and *Coryne- bacterium* spp, while *Enterobacteriaceae* were correlated with better embryo quality. It should be noted that no other factors were taken into account, such as oocyte quality (with preimplantation genetic testing or oocyte donation). Also, embryo quality was assessed based on morphologic criteria on cleavage stage. Studying embryos in blastocyst stage and the use of time-lapse technology would be interesting to better understand the impact on embryo development. In addition, major ART outcomes other than embryo quality have not been addressed so far, such as implantation, clinical pregnancy, ongoing pregnancy, miscarriage and live birth rates.

Given the paucity of studies, their small samples and the inconsistency of their results, it seems quite premature to draw any conclusion on the possible influence of microbiota on seminal quality and male fertility.

The most important limitations of this review is the paucity of data and the fact that most of current knowledge is based on culture and PCR based methods, which narrow a lot the range of bacteria studied so far.

The sample size of most of the studies is small. Samples ranged from 15 to 118 individuals. This may not only limit the power of the studies and the ability to draw conclusions, but also eventually lead to erroneous and biased information.

There is some variation between groups concerning variables studied (eg, some studied richness and diversity of species, other different bacteria) and the level of classification of bacteria used (including within the same study, comparing phyla, classes, genera and species).

Criteria of inclusion and exclusion are not always well defined, in particular concerning medical history and recent use of antibiotics.

The methodology of sample collection was not always well reported, some groups do not describe the aseptic conditions and precautions taken in order to avoid contamination during this step.

There was also considerable variation between the techniques used to sequence bacteria. Researchers used different kits, targeting different hypervariable regions and using different background databases.

The only study focusing resulting embryo quality did not take into account other factors, such as oocyte quality.

In the end, the main limitation of this review and the set of studies included is the inconsistency of their results, sometimes contradictory, which precludes the drawing of any conclusions.

As a conclusion, human semen has its own microbiota, which may eventually play a role on male fertility. However, until date, very few studies have addressed the potential effect of human seminal microbiota and sperm quality, based on whole sequencing of seminal flora with NGS. Additionally, the results of different studies are varied and divergent. In a near future, further and larger studies are need the clarify this matter. Also, studies assessing embryo quality until blastocyst stage and based on time-lapse technology may provide more information about the impact of seminal microbiota on every step of early embryo development. Last but not least, it's important that future studies specifically address the impact of seminal microbioma on ART clinical outcomes, which must be clearly and uniformly defined.

Thus, it seems premature to draw conclusions on this matter.

## Acknowledgements

None.

## Conflicts of interests

The authors have no conflict of interests to report.
